# Affirmative action, critical mass, and a predictive model of undergraduate student body demographics

**DOI:** 10.1371/journal.pone.0250266

**Published:** 2021-05-12

**Authors:** Daniel P. Maes, Julia Tucher, Chad M. Topaz

**Affiliations:** 1 Department of Mathematics and Statistics, Williams College, Williamstown, MA, United States of America; 2 Department of Mathematics, University of Michigan, Ann Arbor, MI, United States of America; 3 Institute for the Quantitative Study of Inclusion, Diversity, and Equity, Williamstown, MA, United States of America; University of Vermont, UNITED STATES

## Abstract

Black and Latinx students are underrepresented on most public university campuses. At the same time, affirmative action policies are controversial and legally fraught. The Supreme Court has ruled that affirmative action should help a minoritized group achieve a *critical mass* of representation. While the idea of critical mass is frequently invoked in law and in policy, the term remains ill-defined and hence difficult to operationalize. Motivated by these challenges, we build a mathematical model to forecast undergraduate student body racial/ethnic demographics on public university campuses. Our model takes the form of a Markov chain that tracks students through application, admission, matriculation, retention, and graduation. Using publicly available data, we calibrate our model for two different campuses within the University of California system, test it for accuracy, and make a 10-year prediction. We also propose a coarse definition of critical mass and use our model to assess progress towards it at the University of California-Berkeley. If no policy changes are made over the next decade, we predict that the Latinx population on campus will move towards critical mass but not achieve it, and that the Black student population will decrease, moving further below critical mass. Because affirmative action is banned in California and in nine other states, it is worthwhile to consider alternative policies for diversifying a campus, including targeted recruitment and retention efforts. Our modeling framework provides a setting in which to test the efficacy of affirmative action and of these alternative policies.

## Introduction

*Affirmative action* policies are those designed to increase representation of minoritized identity groups in spheres where they have faced historical exclusion or discrimination. The identity groups in question typically pertain to gender or race/ethnicity, and common spheres of exclusion include employment, housing, and education [1]. Affirmative action has been at the forefront of the American social consciousness in recent decades. Proponents see affirmative action as necessary to pave the way for equality, while opponents view it as reverse discrimination [2]. In the United States, the debate over affirmative action has been ongoing since the policy’s inception in 1961 [3].

Sixty years later, important questions remain, two of which motivate our work. First, how can we know the effect that an affirmative action policy will have? To date, affirmative action policy assessment overwhelmingly uses retrospective data analysis [4]. That is to say, the most common way to assess a policy has been to implement it and then gather data for several years. One then uses statistical tools to assess whether or not a demographic shift has occurred, and if it is attributable to affirmative action. Policymakers might tweak their policies, wait to see the effect, and repeat, resulting in a lengthy, iterative process.

Our second motivating question is: what does it mean for an affirmative action policy to be successful? As we will later explain, affirmative action should help a particular minoritized group achieve a *critical mass* of representation. While the idea of critical mass is now used in legal proceedings and in policy decisions, the term remains ill-defined. We will propose a coarse metric that could serve as a lower bound on critical mass. Our definition is specific enough to be operationalizable but flexible enough to allow institutional variation. It is crucial to keep in mind that the ultimate goals of affirmative action should be to increase equity and diversity. Critical mass is not an end unto itself, but nonetheless, it is a tool that campuses can use in assessing diversity. Our proposed definition is especially suited for a quantitative modeling framework.

Our work addresses the aforementioned questions by creating a tool for prospective rather than retrospective analysis. We build a forecasting model in the form of a mathematical object known as a Markov chain. We then validate the model and use it to predict racial/ethnic demographics on public campuses. An advantage of a predictive modeling approach is that it provides a test bed for policy experimentation in situations where, as mentioned before, the actual experiment would take years to assess. Our modeling framework allows us not only to predict the effect of various affirmative action admissions policies, but equally, of other policies that might diversify a student body and yet that are less legally contentious. For instance, an educational institution could ask a question such as “if we increase by a few percentage points the probability that Black students accept our admissions offers, what effect might we expect on the overall demographic make-up of the student body in coming years?” and use our model to make a prediction.

More specifically, our study aims to create a predictive model of racial/ethnic demographics in the student bodies of undergraduate programs at public colleges and universities. We use the University of California as our case study because of the large number of students they serve, because of their data transparency practices, and because, as we will explain, the state of California has played a key role in the history of affirmative action. Additionally, University of California schools do not practice race-conscious admissions due to legal limitations. Thus, we can assess the importance of recruitment for underrepresented groups throughout the admissions process in lieu of accounting for race/ethnicity in the admissions process. Still, our model and methods could be straightforwardly applied to other institutions, and to axes of diversity other than race/ethnicity.

The rest of this paper is organized as follows. In Legal History, we provide a review of selected United States Supreme Court cases and California laws that contribute to our understanding of affirmative action and critical mass. In Mathematical Model, California Law, we construct our Markov chain description of the pipeline for college application, admission, matriculation, retention, and graduation. We then calibrate the model using publicly available data from the University of California and validate it by using historical measurements. In Model Predictions, we use the model to predict the racial/ethnic makeup of the undergraduate student body in coming years at the University of California-Berkeley (UCB) and the University of California-Los Angeles (UCLA). Without policy changes, the campus demographics of minoritized groups at UCB will remain largely unchanged over the next ten years, with a slight increase in the proportion of Hispanic/Latinx students and a decrease in the proportion of Black students. For UCLA, we predict increases in the proportion of Hispanic/Latinx students and, to a lesser degree, Black students. Finally, in Critical Mass, we introduce our own definition of the term, and apply it to UCB campus data. If no policy changes are made over the next decade, we predict that the Latinx population on campus will move towards critical mass but not achieve it, and that the Black student population will decrease, moving further below critical mass.

## Legal history

### United States Supreme Court

In *Regents of the University of California v. Bakke*, 438 U.S. 265 (1978), the Supreme Court considered the case of Alan Bakke, a White man who was twice rejected from medical school at the University of California-Davis (UCD). At the time, UCD reserved 16 out of 100 of their admitted spots for qualified minorities. Bakke’s admissions ratings were higher than any of the minority students’ in both years of applying. Bakke claimed he was denied admissions unfairly, and solely based on his race. The court ruled 5-4 in favor of Regents that Title VI of the Civil Rights Act of 1964 does not prohibit race-based admissions programs, and that the Equal Protection Clause of the 14th amendment permits race to be a factor in admissions. However, they also ruled 5-4 that the Equal Protection Clause does prohibit the use of racial quotas of the type UCD used. Therefore, the court instructed the university to admit Bakke.

Another landmark case was *Gratz v. Bollinger*, 539 U.S. 244 (2003). When admitting undergraduate students, the University of Michigan (UM) considered grades, test scores, relationship with alumni, geography, leadership qualities, and more. UM scored each applicant on a 150 point scale, where 100 points guaranteed admission. They also considered race/ethnicity in their decisions, adding 20 points for students whom they considered to be from underrepresented minority groups. In 1995, White in-state students Jennifer Gratz and Patrick Hamacher were both rejected for admission. In 1997, they filed a class action lawsuit against the university on the grounds of racial discrimination. The court decided 6-3 in favor of the students that the university’s policy was not narrow enough to meet *strict scrutiny*. Strict scrutiny is a standard used to determine the constitutionality of a policy. To pass strict scrutiny, a policy must serve a compelling government interest and be narrowly tailored. The court found UM’s policy to fail strict scrutiny because it assumed that every applicant from a specific underrepresented minority group was from a similar background. This decision helped establish the use of strict scrutiny for evaluating affirmative action policies in public higher education.

*Grutter v. Bollinger*, 539 U.S. 306 (2003) also involved UM. Barbara Grutter, a White in-state student, applied to UM’s law school in 1997. Despite a high undergraduate gradepoint average and high admissions exam scores, she was denied admission. The law school had a stated policy of using race/ethnicity in their admissions decisions because having a *critical mass* of minority students is in the interest of the state. The court sided with UM, finding that their highly individualized application review meant that no acceptance was based on one sole factor, including race. The verdict upheld the verdict of *Regents v. Bakke*, namely, that there is a compelling interest in achieving a diverse student body. Pivotally, though, the court clarified for the first time that the goal of critical mass does not equate to a quota system. That is, a highly individualized use of race/ethnicity in admissions is constitutional.

The most recent landmark case for affirmative action in higher education is *Fisher v. University of Texas*, 579 U.S. __ (2016), commonly known as *Fisher II*. In Texas, a policy called the Top Ten Percent Plan guarantees admission to the University of Texas (UT) for any public high school student graduating in the top ten percent of their class. In-state applications that do not qualify under this policy are evaluated according to a holistic process which does include consideration of race/ethnicity. In 2008, White in-state student Abigail Fisher, who did not qualify for admission to UT under this policy, had her application rejected. Fisher sued the university, arguing that the use of race as an admissions consideration was a violation of the Equal Protection Clause. The court ruled 4-3 in favor of UT, finding that the university was allowed to use race/ethnicity as a factor in admission policies on condition that they would study how diversity was being achieved or maintained on campus. Similarly to in *Grutter v. Bollinger*, the university’s approach was upheld because it used a rather individualized assessment of an application, using race as one factor, and because there were no workable alternatives for achieving diversity, already found to be a compelling interest.

Overall, the appropriate means of implementing affirmative action in undergraduate admissions remain ill-defined. From *Regents v. Bakke*, racial/ethnic quotas were deemed unconstitutional, and so the mechanisms of affirmative action needed to become broader in order for policies to persist. Eventually, the judiciary arrived at the term used in the most recent court cases, namely, critical mass. In *Fisher II*, the court stated that “critical mass is neither some absolute number of African-American or Hispanic students nor the percentage of African-Americans or Hispanics in the general population of the State…and [the] term remains undefined.” This lack of specificity is a challenge of contemporary implementations of critical mass. Any undergraduate institution can dictate its own interpretation of critical mass, and since diversity is already considered a compelling government interest, almost any definition of critical mass will pass the standard of strict scrutiny.

In summary, as legal precedent has forced affirmative action policies to become more ill-defined, the effectiveness of those policies has become more difficult to assess. While the Supreme Court has recognized that diversity is beneficial to college campus, it is difficult to know if specific admissions practices and policies effectively support the promotion of diversity. The idea of critical mass exists as a tool to help universities achieve the goal of diversifying campus. Critical mass has, to date, lacked an operationalizable definition. Later, we will propose one that is relevant for quantitative modeling efforts. First, we narrow our attention to the State of California in order to elucidate the context for our modeling study.

### California law

Outside of federal policies and decisions, states and institutions have a say in how and to what extent their public systems use affirmative action policies. Because of our own focus on the University of California system, we now review key legal developments regarding affirmative action in public undergraduate admissions in California.

In 1995, the Regents of the University of California passed a resolution called SP-1 which eliminated the use of race, ethnicity, and gender in admissions decisions for institutions in the UC system. A year later, California voters amended their state constitution by passing a ballot initiative called Proposition 209. The amended constitution prohibits state and local agencies from giving preferential treatment to individuals or groups on the basis of their race, sex, ethnicity, or national origin in public education, employment, or contracting. As a result, schools in the UC system are not allowed to use affirmative action in their admissions policies [5].

Three years after Proposition 209 passed, California implemented a state-wide policy that guaranteed admissions to UC for top students in public schools. Under this policy, a student was guaranteed admission to (at least) one school in the UC system if they were in the top four percent of a California public high school’s graduating class or were in the top four percent of graduating students statewide. Students admitted under the “Four Percent Plan did not get to choose the institution to which they were admitted. The plan remains in place, though in 2012 it was expanded from four percent to nine percent [5].

Meanwhile, in 2001, the Regents voted to rescind SP-1 and they replaced it with Regents Policy 4401. This policy affirmed that all students would be treated equally in the admissions process regardless of their race, sex, color, ethnicity, or national origin. The policy also specified that each campus should seek to enroll a student body which demonstrates a high academic achievement or talent level, as well as encompassing the broad diversity of backgrounds represented in the state of California [6]. Though the Regents rescinded SP-1, the passage of Proposition 209 several years earlier still prohibits affirmative action policies at UC institutions. However, there has been movement in California to attempt to repeal Proposition 209. In particular, California Proposition 16 would have reverted the state constitution, thus allowing the affirmative action policies to be used in undergraduate admissions. In November 2020, though, California voters rejected Proposition 16 and hence affirmative action policies remain banned in California. Therefore, it is useful to focus on factors that influence campus demographics other than the acceptance rates of various racial/ethnic groups. For example, an admissions office attempting to recruit more heavily from certain demographic groups would not be in violation of bans on affirmative action. The mathematical model we will construct will enable the assessment of the impact of such practices.

## Mathematical model

We now construct a model for predicting racial/ethnic demographics in a four-year undergraduate program. We will later apply this model to UCB and UCLA.

From the start, it is important to keep in mind several limitations of our modeling approach. Mirroring the limitations of the public data from which we compute model parameters, our model only accounts for students who are Asian American, Black, Hispanic/Latinx, or White. The modeling framework does not account for multiple racial/ethnic identities, nor any identities other than the aforementioned four. As for individuals who are Native American, Alaska Native, Pacific Islander, Native Hawaiian, Middle Eastern, multi-racial/ethnic, and others, their unfortunate omission from the model is related to issues of data availability and/or sample size. Future work should attempt to utilize more complete data that would remedy this erasure.

In addition to the data limitations above, a second set of limitations pertains to modeling assumptions that we make for simplicity. We account neither for international students nor students transferring into or out of the campuses we model. Instead, we focus on students entering a U.S. campus directly from a U.S. high school. Future models could incorporate other routes to matriculating at an institution. Similarly, we do not account for various possible complex paths through an undergraduate program, including students who take longer than six years to graduate. Our model should be viewed as a first step towards predictive modeling of undergraduate demographics. Future iterations of the model might relax some of the simplifying assumptions we have made here.

The remainder of this section is organized as follows. In Markov Chain Model Construction, we build our basic modeling framework, which tracks one demographic group of high school graduating class year through college application, admission, matriculation, retention, and graduation. In Markov Chain Transition Rates, we explain how entries in our Markov chain transition matrix can be deduced from publicly available data. In Inferring Model Parameters, we forecast this public data ahead in time in order to be able to specify Markov chain transition rates in future years. In Model Simulation and Validation, we detail how to combine results from multiple demographic groups and multiple high school graduation class years in order to predict overall student demographic proportions on a campus. We also explain how to generate 95% confidence intervals on our model output, where these intervals arise from uncertainty in calculation of the Markov chain transition rates. Finally, we train and test our model on historical data to provide evidence of the efficacy of the modeling approach.

### Markov chain model construction

Students enter our modeling framework as graduating high school seniors who apply and are accepted to a post-secondary institution. Then, they choose whether or not to attend the institution. If they enroll, they graduate in four to six years, though there is a possibility of dropping out after each semester. We model this process with a *Markov chain*, a memoryless model that describes the evolution of the probability of the system being in any particular state. For a review of Markov Chains, see [7]. In our model, there are 24 states representing students’ progression through college: a high school student applying, graduating, and being accepted (states 1—3); a college student in their first, second, third, fourth, or fifth year during the fall term, spring term, and in between years (states 4—18); a sixth-year college student in their fall or spring term (states 19—20); a college student graduating after four, five, or six years (states 21—23); and a college student dropping out (state 24). Because Markov chains describe probabilities, and because we know the sizes of relevant populations (applicant pools, student bodies, and so forth) we can also calculate expected values of the number of students in various states. In fact, our discrete Markov chain model is a special case of a more general class of models, namely Leslie matrix population models, typically used to model age structure in biological populations [8]. Therefore, it is as natural to specify the model’s state as population counts as it is to specify a probability distribution.

Given the set of all states *S* = {*s*_1_, *s*_2_, …, *s*_24_}, the probability of moving from state *s*_*i*_ to *s*_*j*_ is *p*_*ij*_; these quantities are called *transition probabilities*. Once we specify the transition probabilities, we construct a *transition matrix* **P**: a 24 × 24 matrix where the *ij*th entry is the transition probability *p*_*ij*_. One represents the state of the system as a vector and progresses the Markov chain via multiplication by **P**. Conveniently, the matrix **P**^*n*^ gives the probability of a student moving from state *s*_*i*_ to state *s*_*j*_ in *n* steps. For our model, each step represents a calendar time of four months.

Our model is an *absorbing Markov chain*, meaning that for some states in the model, the probability of leaving that state is zero, and equivalently *p*_*ii*_ = 1 and *p*_*ij*_ = 0 for all *i* ≠ *j*. These states are *absorbing states*, and all non-absorbing states are *transient states*. Our model is an absorbing Markov chain because after a student graduates or drops out, they do not re-enroll at the college. We split the states into two distinct sets *T* = {*s*_1_, *s*_2_, …, *s*_*t*_} and *F* = {*s*_*t*+1_, *s*_*t*+2_, …*s*_*t*+*f*_}, where *T* is the set containing all *t* transient states and *F* is the set containing all *f* absorbing states. The original set of states is *S* = *T* ∪ *F* and contains all *t* + *f* = 24 states in the Markov chain.

For our analysis, we put the transition matrix **P** into *canonical form*

$$P=\begin{array}{cc} & \begin{array}{cc} T & F \end{array}\\ \begin{array}{l} T\\ F \end{array} & \left(\begin{array}{cc} Q & R\\ 0 & I \end{array}\right) \end{array}$$ (1)

where **Q** is a *t*-by-*t* matrix, **R** is a nonzero *t*-by-*f* matrix, **0** is the *f*-by-*t* zero matrix, and **I** is the *f*-by-*f* identity matrix. Now consider the form of the matrix **P**^*n*^, which owing to the upper block triangular form of **P** is:

$$P^{n}=\begin{array}{cc} & \begin{array}{cc} T & F \end{array}\\ \begin{array}{l} T\\ F \end{array} & (\begin{array}{cc} Q^{n} & *\\ 0 & I \end{array}). \end{array}$$ (2)

Here, the asterisk is a placeholder for a *t*-by-*f* matrix. It is a standard calculation to show that due to the absorbing nature of our Markov Chain, **Q**^*n*^ → **0** as *n* → ∞, meaning that that over time, the probability of a student moving into a transient state approaches zero. This limit reflects that in our model, the college process eventually terminates for every student.

Prior to calculating initial conditions and transition rates, we describe the states of the Markov chain and the transitions between them. As mentioned earlier, our model is comprised of 24 states. For convenience, we use shorthand to denote each one. For the 23 states that are not drop-out, we pair a number from zero through six to describe the academic year (where zero represents senior year of high school) and a letter from the set {*A*, *B*, *C*, *G*} to correspond to the term or standing within that year. Specifically, *A* represents the fall term, *B* represents the spring term, *C* represents the summer term (or the transition from one year to the next), and *G* corresponds to graduation. For example, a high school senior in the summer before college is in state 0*C*, a third-year college student in their fall term would be in state 3*A*, and a student who graduated after six years would be in state 6*G*. We denote the remaining stage as *DNF*, meaning the student did not finish their college education. Thus, we have the complete set of states

$$\begin{array}{c} S=\{0A,0B,0C,1A,1B,1C,\ldots ,5A,5B,5C,6A,6B,4G,5G,6G,\text{DNF}\}. \end{array}$$ (3)

As mentioned in our discussion of modeling limitations, we assume that after graduating or dropping out, a student neither re-enters the applicant pool nor re-enrolls in college. We separate the states into transient and absorbing sets:

$$\begin{array}{c} \begin{array}{c} T=\{0A,0B,0C,1A,1B,1C,2A,2B,2C,3A,3B,3C,4A,4B,4C,5A,5B,5C,6A,6B\},\\ F=\{4G,5G,6G,\text{DNF}\}. \end{array} \end{array}$$ (4)

**Fig 1:**
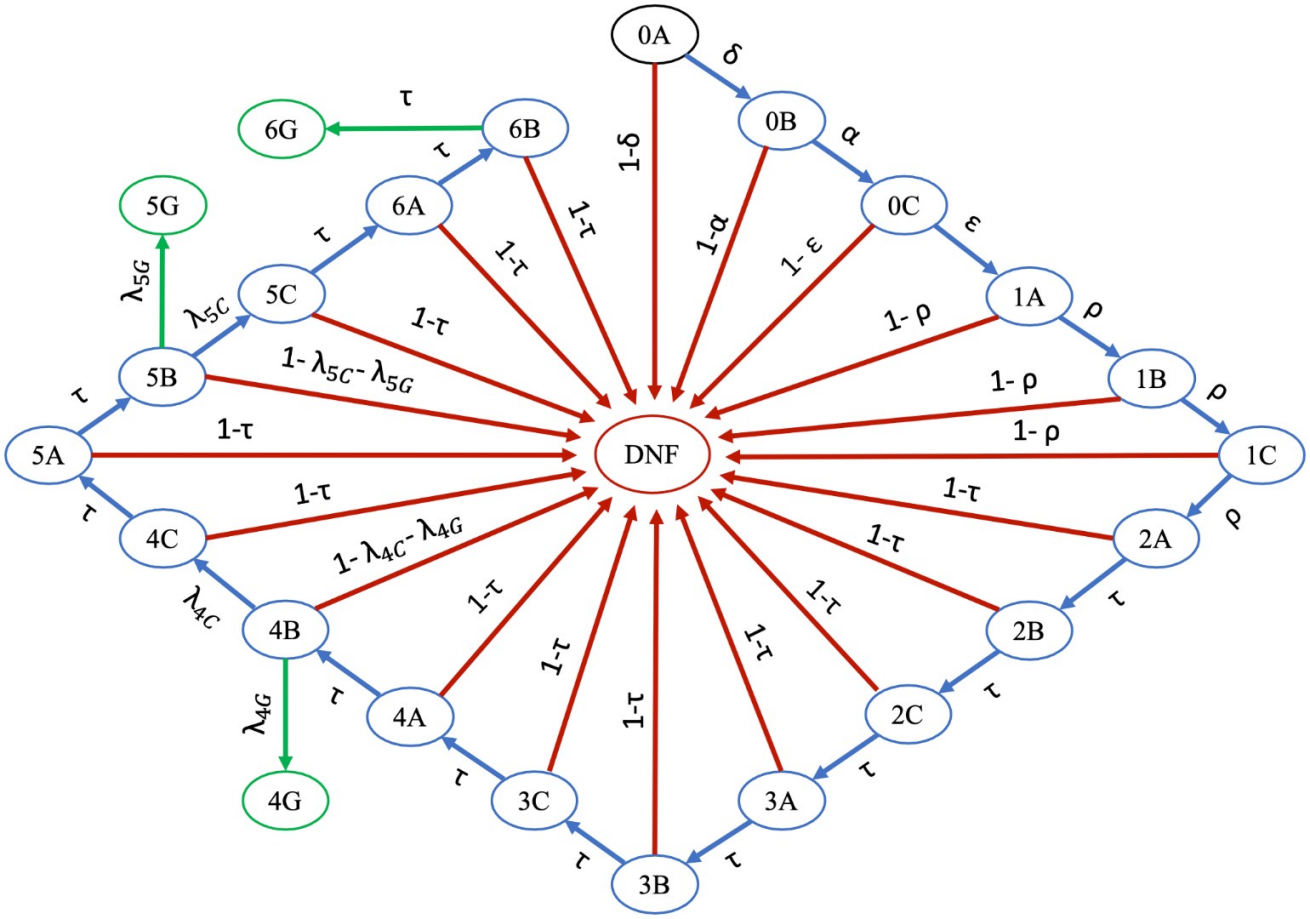
Markov chain model for predicting undergraduate student body demographics. To label nodes, we pair a number from zero through six to describe the academic year (where zero represents pre-college year) and a letter from the set {*A*, *B*, *C*, *G*} to correspond to the term or standing within that year. *A* represents the fall term, *B* represents the spring term, *C* represents the summer term (or the transition from one year to the next), and *G* corresponds to graduation. The state *DNF* represents students who did not obtain an undergraduate degree. A student begins in state 0*A* and then moves through the graph according to the probabilities on each directed edge. Blue nodes represent transient states, green nodes represent the absorbing states of graduating, and red nodes represent the absorbing state of not finishing. Arrows are labeled with the transition probabilities between states.

We can represent the Markov chain model as a graph where nodes are the states of the model and directed weighted edges represent the transition rates between states; see Fig 1. A student begins in state 0*A* and then moves through the graph according to the probabilities on each directed edge. Blue nodes represent transient states, green nodes represent the absorbing states of graduating, and the red node represents the absorbing state of dropping out.

### Markov chain transition rates

We now specify the transition matrix **P** that governs our model by deriving the transition probabilities between each state. In particular, we define the transition probabilities in terms of quantities that we can infer from public data. These publicly available quantities are application, acceptance, and enrollment counts, first-year retention rates, and rates for four-, five-, and six-year graduation. Below, we construct the transition probabilities in an abstract framework, without attaching numerical values to them. In the next subsection, we will assign numerical values in order to specify model parameters for specific institutions in specific years.

We introduce one piece of notation to simplify our discussion below. We use a right arrow to denote the probability of transition from one state to another, where the two states are not necessarily adjacent in the Markov chain. That is to say, we can use the right arrow to construct probabilities not just for simple transitions, but for compound ones. For example, we express the first-year retention rate as 1*A* → 2*A*, the probability that a student in stage 1*A* passes through stages 1*B* and 1*C* and ends in stage 2*A*. As the model is a directed acyclic graph, our compound transitions have exactly one path.

We can now specify our transition rates in terms of accessible data. First, we use admissions data to find transition probabilities for pre-college stages. Define *δ*, the probability of a graduating high school student applying to the college, as

$$\begin{array}{c} \delta =0A\to 0B=\frac{\;\text{number}\;\text{of}\;\text{applicants}}{\text{number}\;\text{of}\;\text{high}\;\text{school}\;\text{graduates}}. \end{array}$$ (5)

Define *α*, the probability of being accepted to the college after applying, as

$$\begin{array}{c} \alpha =0B\to 0C=\frac{\;\text{number}\;\text{of}\;\text{acceptances}}{\text{number}\;\text{of}\;\text{applicants}}. \end{array}$$ (6)

Lastly, define *ϵ*, the probability of an accepted student matriculating, as

$$\begin{array}{c} \epsilon =0C\to 1A=\frac{\text{number}\;\text{of}\;\text{enrollees}}{\text{number}\;\text{of}\;\text{acceptances}}. \end{array}$$ (7)

Now we focus on the progression through college. Let *v* represent the rate of first-year retention, that is, the compound rate of transition 1*A* → 2*A*. Our model requires specification of the probabilities for 1*A* → 1*B*, 1*B* → 1*C*, and 1*C* → 2*A*. As a simplifying assumption, we take these to be equal because we only have access to yearly retention statistics. Therefore, each of these transitions has probability $\rho =\sqrt[{3}]{v}$.

We have treated transitions during the first year separately above because retention data is publicly available only for the first year. We now consider transitions for the remaining years of college, that is, states 2*A* through 6*G*. Let *τ* be the probability of continuing from one term to the next term or to graduation; stated differently, *τ* is the probability of not dropping out. We refer to *τ* as the *basic transition rate*. This is the per-term retention rate for states 2*A* through 4*B* because we assume that a student cannot graduate in fewer than four years. On the other hand, the probability 4*B* → 4*C* is not equal to *τ* because the student might graduate. In this case, *τ* is the sum of the probabilities that a student continues from 4*B* to 4*C* or to 4*G*. That is to say, *τ* accounts for a student continuing on in school or graduating. We have a similar situation for 5*B* → 5*C*. As our model has a six-year graduation cap, students cannot continue to enroll after six years. Thus, 6*B* → 6*G* = *τ*.

As we will discuss later, from public data, we know the rates of first-year retention, as well as four-, five-, and six-year graduation. Our model in Fig 1 contains five unknown transition rates *τ*, λ_4*C*_, λ_5*C*_, λ_4*G*_, λ_5*G*_. We now specify a system of equations that will let us solve for these model parameters in terms of the known public data. First, we note that there are seven transitions made between 2*A* and the state preceding four-year graduation, 4*B*, and thus 2*A* → 4*B* = *τ*^7^. After completing four years of college, students graduate with probability λ_4*G*_. We can thus express the four-year graduation rate, denoted *γ*_4_, as

$$\begin{array}{c} \gamma_{4}=v\tau^{7}\lambda_{4G}. \end{array}$$ (8)

Next, because we assume a constant drop-out rate of 1 − *τ*, we express continuation after four years to either a fifth year or graduation as

$$\begin{array}{c} \tau =\lambda_{4G}+\lambda_{4C}. \end{array}$$ (9)

The five-year graduation rate *γ*_5_ is cumulative. That is, it includes those that graduate in five or fewer years, so we express it as the sum of the four-year graduation rate and the probability of a student completing five years and then graduating:

$$\begin{array}{c} \gamma_{5}=\gamma_{4}+v\tau^{9}\lambda_{4C}\lambda_{5G}. \end{array}$$ (10)

The basic transition *τ* rate also represents the probability of continuation after five years to either graduation or a sixth year, and so

$$\begin{array}{c} \tau =\lambda_{5G}+\lambda_{5C}. \end{array}$$ (11)

Finally, making an argument similar to that for *γ*_5_ above, we express the six-year graduation rate *γ*_6_ as the sum of the five-year graduation rate and the probability of a student completing college in exactly six years and then graduating. Thus,

$$\begin{array}{c} \gamma_{6}=\gamma_{5}+v\tau^{12}\lambda_{4C}\lambda_{5C}. \end{array}$$ (12)

Through straightforward algebraic manipulation, we combine (8)–(12) into a single polynomial equation in terms of *τ*:

$$\begin{array}{c} 0=v\tau^{14}-\gamma_{4}\tau^{6}+\left(\gamma_{4}-\gamma_{5}\right)\tau^{3}+\left(\gamma_{5}-\gamma_{6}\right). \end{array}$$ (13)

Because *τ* is meant to be a probability and should be uniquely determined, we need to show that the polynomial *f*(*τ*) = *vτ*^14^ − *γ*_4_*τ*^6^ + (*γ*_4_ − *γ*_5_)*τ*^3^ + (*γ*_5_ − *γ*_6_) has exactly one real root on the interval (0, 1).

*Proof*. Note that the coefficients in *f*(*τ*) are on the interval [0, 1] because they involve rates of graduation and first-year retention. As a polynomial with real coefficients, *f*(*τ*) is continuous on [0, 1]. At the left endpoint of this interval, we have

$$\begin{array}{c} f\left(0\right)=\gamma_{5}-\gamma_{6}<0, \end{array}$$ (14)

as more people graduate in six years than in five. At the right endpoint of the interval, we have

$$\begin{array}{c} f\left(1\right)=v-\gamma_{6}>0, \end{array}$$ (15)

since, by definition, fewer people graduate in six years than complete their first year of college. Since the function is continuous and has values with opposite signs at the endpoints, then by the Intermediate Value Theorem, there must be at least one root of *f* on (0, 1). Finally, by Descartes’ Rule of Signs for polynomials, there is at most one positive real root. Thus, there exists exactly one positive real root on (0, 1) and we refer to it as our basic transition rate.

With *τ* determined (say, using a numerical solver), we substitute into (8)–(11) to find the remaining parameters λ_4*G*_, λ_4*C*_, λ_5*G*_, λ_5*C*_. With transition rates in the model now specified in terms of accessible data, we compile them into our transition matrix. Recall that when in canonical form, the two key block matrices in **P** are **Q** and **R**; see (1). Then

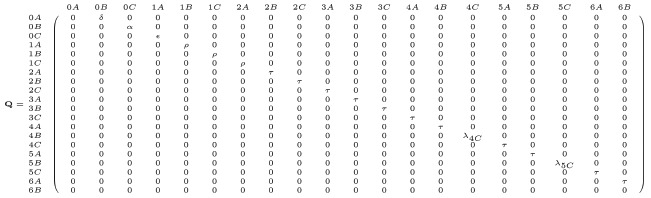
(16)
and

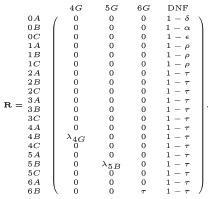
(17)

### Inferring model parameters

We now calibrate our model to make predictions for UCB. To obtain numerical values for the transition probabilities, we use national high school graduation counts and UCB admission and graduation statistics. We will forecast data for the next 10 years using data gathered from the National Center for Education Statistics (NCES) [9], the UC undergraduate admissions summaries [10], and the UC records on graduation and retention rates [11]. Our model will provide a predicted demographic breakdown of UCB over that same time period. As each type of data differs across racial/ethnic groups, we will construct four Markov chains, each representing the academic progression of students from one racial/ethnic group.

**Fig 2:**
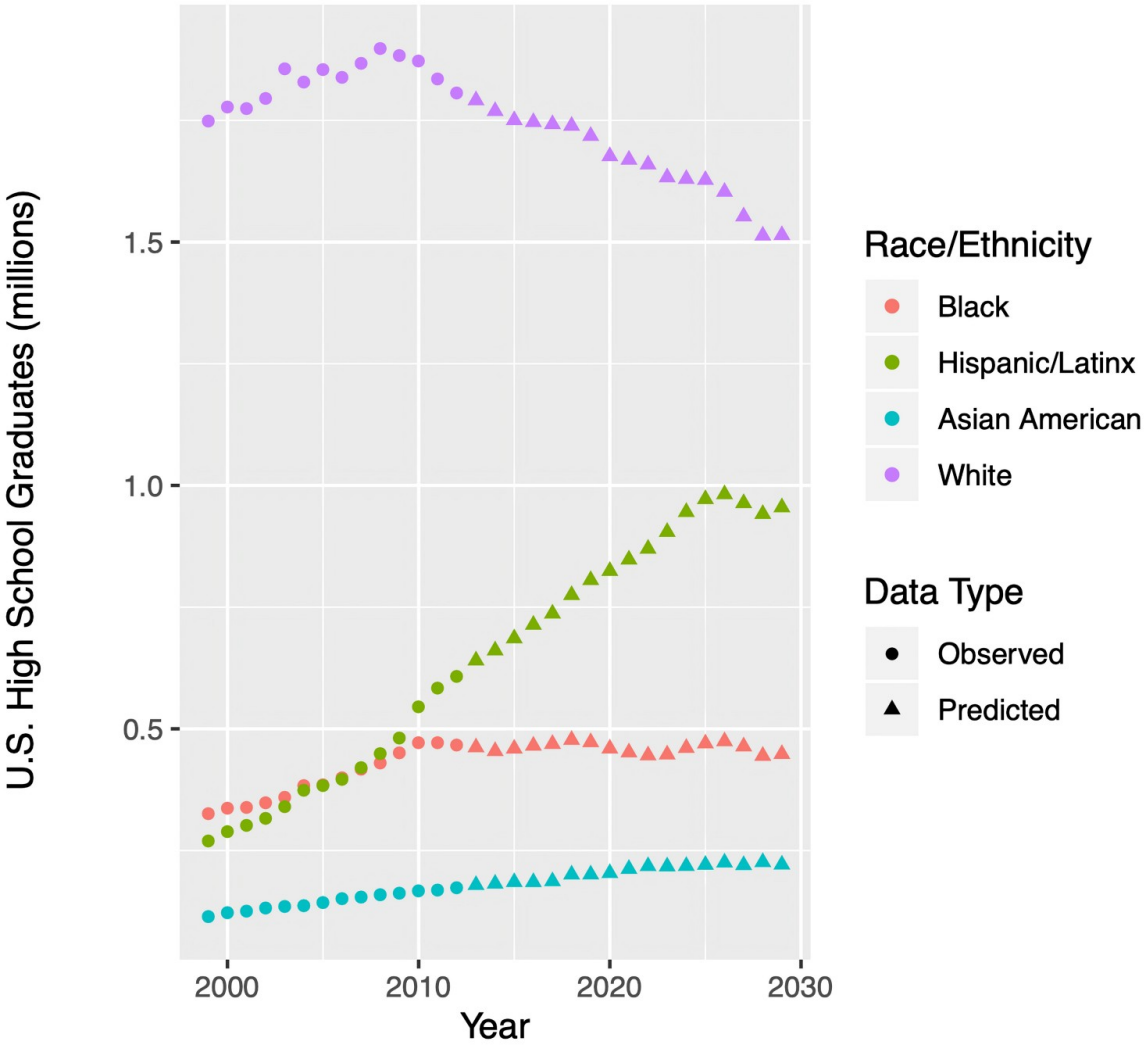
U.S. high school graduates by year and racial/ethnic group. Observed and predicted counts of public high school graduates in the U.S. between 1999 to 2029, in millions, according to data from the NCES [9]. The graph shows measured data through 2013 (circles) as well as NCES projections after 2013 (triangles).

The input for each Markov chain is a vector with one nonzero value: the count of graduating high school students of the specified racial/ethnic group that year. Fig 2 shows NCES data on the number of graduating high school seniors per year in the Unites States, broken down by four racial/ethnic groups. The data spans 1999 to 2029. The values from 1999—2013 are observed values, while those after 2013 are forecast by NCES. In Fig 2, the NCES projected values are depicted as triangles. Our model will use the NCES projections from 2015-2029. We also use these counts in conjunction with applicant counts to help determine the rate of application, *α*.

The UC undergraduate admissions summaries include application, acceptance, and matriculation counts through 2019. While data is available as far back as 1994, we only use data from 1998 onwards. Affirmative action was discontinued at UCB in 1997, and we wish to study UCB’s current, and not prior, admissions policies. Using this data, we will make our own predictions spanning the next ten years.

**Fig 3:**
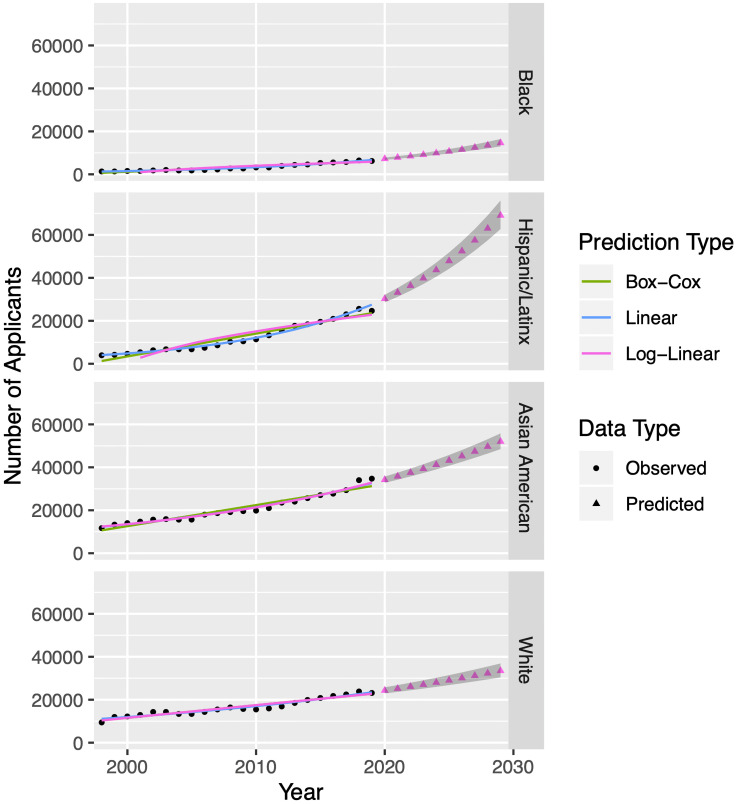
Regression models for applicant data from University of California-Berkeley. To model applicant data in each of four racial/ethnic groups from 1998—2019 (black dots) we apply three potential regression models, namely optimal Box-Cox (green), linear (blue) and log-linear (pink) to observed data from the University of California system [10]. We perform a standard residual analysis on each model in order to choose the best one. For the parameters depicted above, the best model happens to be log-linear for each. Table 1 provides our chosen fits for all applciation, acceptance, and enrollment parameters. Using our chosen models, we forecast data from 2020—2029 (triangles). The 95% confidence intervals on predicted values appear as gray bands. We will use these intervals later to generate 95% confidence intervals on the output of our Markov chain model. While the figure above shows fits for applicant counts, we follow a similar procedure for acceptance counts and enrollment counts; see Table 1. Note: if there is no optimal Λ for the Box-Cox transformation, the regression procedure defaults to linear. Hence, the linear and Box-Cox models sometimes overlap.

We will compare three different regression models to choose a method of forecasting for each of the twelve subsets of this admissions data set. These twelve subsets comprise one for each of the three admission statistics for four racial/ethnic groups. Fig 3 visualizes the annual applicant count at UCB by racial/ethnic group and compares the performance of linear models on three transformations of the dependent variable. UCB data appears as black dots and predicted data appears as triangles in a color corresponding to the regression model selected.

More specifically, we perform a linear regression (blue), log-linear regression (pink), and linear regression after optimal Box-Cox transformation (green) and plot them from 1998 through 2019 to compare their performances on the observed data. We perform a standard residual analysis on each model in order to choose the best one. For applicant counts across all four racial/ethnic groups, fitted values generated by log-linear regression align better than linear or Box-Cox regression. Table 1 specifies our chosen models not just for applicant counts, but additionally for acceptances and enrollments. Comparing these regressions across all data sets, log-linear frequently performs as well as or better than linear and Box-Cox, with the exceptions of Black student enrollment and Black and White student acceptances, where the optimal Box-Cox transformation produces a better fit. We will use the best-fitting type of regression to predict each type of UCB undergraduate admissions data for the next 10 years. However, for applicants, acceptances, and enrollments, adding up the predictions for the racial/ethnic groups leads to a sum that is greater than the forecasted value for the total. Thus, after gathering forecast values for each racial/ethnic group, we scale predictions by the forecast total.

**Table 1:** Regression models for applications, acceptances, and enrollment at University of California-Berkeley. Our Markov chain model (see Fig 1) requires specification of the parameters *δ*, *α*, and *ϵ*, which are probabilities derived from counts of applicants, acceptances, and enrollments. These counts must be specified for each year and for each racial/ethnic group. We assess the fit of linear, log-linear, and optimal Box-Cox models on historical data. We choose the preferred model, specified in the table above, and use it to forecast future values. Fig 3 shows various models for application count, corresponding to the top section of the table. The column labeled Λ is an exponent used in the Box-Cox transformation, and thus is relevant only to those fits.

Parameter Type	Racial/Ethnic Group	Best Model	Intercept	Slope	Λ
Applicant Count	Black	Log-Linear	-133.035	0.070	N/A
	Asian American	Log-Linear	-82.556	0.046	N/A
	Hispanic/Latinx	Log-Linear	-168.666	0.088	N/A
	White	Log-Linear	-58.562	0.034	N/A
Acceptance Count	Black	Box-Cox	-4973.058	2.537	0.777
	Asian American	Log-Linear	-51.474	0.030	N/A
	Hispanic/Latinx	Log-Linear	-92.701	0.050	N/A
	White	Box-Cox	-0.491	0.003	-0.144
Enrollment Count	Black	Box-Cox	-15254.672	7.805	1.259
	Asian American	Log-Linear	-42.177	0.025	N/A
	Hispanic/Latinx	Log-Linear	-103.795	0.055	N/A
	White	Log-Linear	-17.921	0.012	N/A

**Fig 4:**
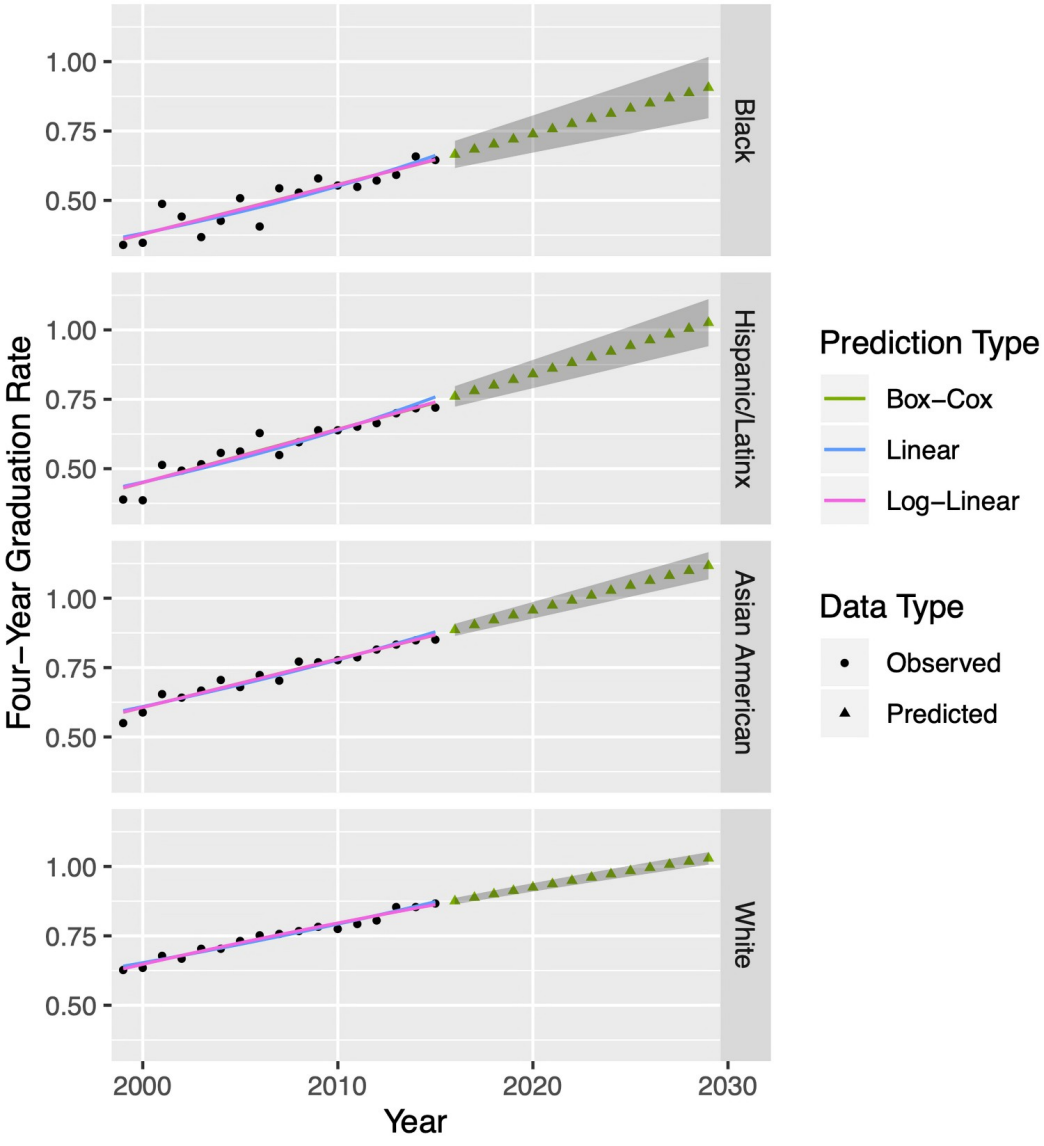
Regression models for four-year graduation rates at University of California-Berkeley. To model four-year graduation rates in each of four racial/ethnic groups from 1998—2015 (black dots) we apply three potential regression models, namely optimal Box-Cox (green), linear (blue) and log-linear (pink) to observed data from the University of California system [11]. We perform a standard residual analysis on each model in order to choose the best one. For the parameters depicted above, the best model happens to be Box-Cox for each. Table 2 provides our chosen fits. Using our chosen models, we forecast data from 2016—2029 (triangles). The 95% confidence intervals on predicted values appear as gray bands. We will use these intervals later to generate 95% confidence intervals on the output of our Markov chain model. While the figure above shows fits for four-year graduation rates, we follow a similar procedure for five-year and six-year graduation rates, as well as first-year retention rates; see Table 2. Note: if there is no optimal Λ for the Box-Cox transformation, the regression procedure defaults to linear. Hence, the linear and Box-Cox models sometimes overlap.

Lastly, we perform a process similar to that described above on data from UCB’s Office of Planning and Development in order to predict first-year retention rate and graduation rates for incoming classes, that is, *ρ*, *γ*_4_, *γ*_5_, and *γ*_6_. All four of these rates once again follow different patterns with respect to racial/ethnic groups, so we will split the data for each parameter accordingly and fit each with the best regression model. As an example, we provide in Fig 4 the observed and predicted four year graduation rates *γ*_4_ for the four racial/ethnic groups. Our methodology projects some of these rates to exceed one, which is impossible, so when used in the model, we cap them to a maximum value of one. Similar to Tables 1 and 2 provides our chosen regression models for each of the four rate parameters across all four racial/ethnic groups.

**Table 2:** Regression models for retention and graduation rates at University of California-Berkeley. Our Markov chain model (see Fig 1) requires specification of the parameters λ_4*G*_, λ_5*G*_, λ_6*G*_, and *ρ*, which are related, respectively, to the four-year graduation, five-year graduation, six-year graduation, and first-year retention rates. These rates must be specified for each year and for each racial/ethnic group. We assess the fit of linear, log-linear, and optimal Box-Cox models on historical data. We choose the preferred model, specified in the table above, and use it to forecast future values. Fig 3 shows various models for four-year graduation rates, corresponding to the top section of the table above. The column labeled Λ is an exponent used in the Box-Cox transformation, and thus is relevant only to those fits.

Parameter Type	Racial/Ethnic Group	Best Model	Intercept	Slope	Λ
First-Year Retention	Black	Linear	-2.616	0.001	N/A
	Asian American	Linear	-0.626	0.001	N/A
	Hispanic/Latinx	Linear	-2.616	0.001	N/A
	White	Linear	-1.954	0.001	N/A
Four-Year Grad Rate	Black	Box-Cox	-31.332	0.015	0.997
	Asian American	Box-Cox	-18.433	0.009	1.924
	Hispanic/Latinx	Box-Cox	-30.317	0.014	0.949
	White	Box-Cox	-21.369	0.010	1.491
Five-Year Grad Rate	Black	Box-Cox	-13.927	0.006	2
	Asian American	Box-Cox	-5.776	0.003	2
	Hispanic/Latinx	Log-Linear	-17.752	0.008	N/A
	White	Box-Cox	-6.275	0.003	2
Six-Year Grad Rate	Black	Box-Cox	-13.409	0.006	N/A
	Asian American	Box-Cox	-5.756	0.002	2
	Hispanic/Latinx	Log-Linear	-11.623	0.005	N/A
	White	Box-Cox	-6.382	0.003	-2

### Model simulation and validation

With our basic Markov chain constructed and input parameters inferred, we proceed to using our model to simulate student body demographics across an institution. To seed our model, we initialize four starting vectors, each of which represents students in one of the racial/ethnic groups. All are high school seniors. Then, we repeatedly multiply this vector by the associated transition matrix to simulate iteration forward, where each multiplication represents forward progression in time by a 4-month time step. Three forward iterations equate to one year. At the end of each year, we record the number of students in each state. Additionally, we then update the transition matrix to reflect the changing application, admission, and graduation rates. To produce overall demographic counts for an institution at the end of year *t*, we add the counts from the Markov chains initiated during years *t*, *t* − 1, *t* − 2, and *t* − 3.

Our model results also include 95% confidence intervals on predictions, where these intervals arise because of the uncertainty of input parameters. For each year of prediction and for each of four demographic groups, our model requires specification of seven parameters, namely the rates of application, acceptance, matriculation, first-year retention, and four-, five-, and six-year graduation, for a total of 28 parameters per year. To make a ten-year prediction, then, requires the specification of 280 parameters. We utilize a Latin hypercube sampling (LHS) method to randomly sample these input parameters [12]. More specifically, we sample uniformly from the 95% confidence intervals associated with our parameter forecasts. We use these parameter samples as inputs to our model. Using the “rule-of-ten,” which suggests ten samples per parameter, we produce 2800 LHS parameter sets and run our model for each. We take the range of model outputs to represent a 95% confidence interval for model predictions.

**Fig 5:**
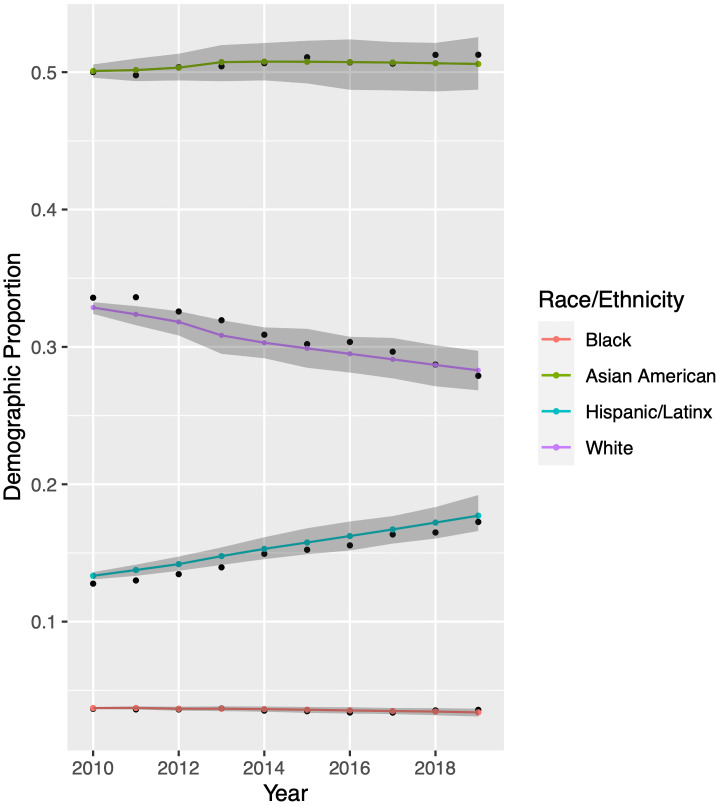
Model validation for University of California-Berkeley. Historical enrollment records appear as black dots. Colored dots are model output obtained when using the parameters forecast in Inferring Model Parameters. We perform an additional 2800 model simulations using parameter sets generated by Latin hypercube sampling. Grey bands indicate the central 95% of these results. Demographic proportions are relative to each other; see Mathematical Model.

Before using our model to make demographic forecasts, we conduct a small data validation study. We seed the model with data from 1998—2009. We then run the model to generate “predictions” for 2010—2019 and compare them to observed data from the same time period. Results for UCB appear in Fig 5. The true, observed demographic proportions at UCB are depicted as black dots and model output appears as colored dots which are connected by line segments to guide the eye. The 95% confidence intervals of the model appear in grey, and we note that the observed demographic proportions mostly fall within these intervals. Overall, our model is quite accurate compared to the observed enrollment records. The model’s confidence interval widens over time, reflecting uncertainty in the forecasting of model parameters; see Figs 3 and 4. Regardless, our predicted proportions of Black, Asian American, Hispanic/Latinx, and White students are accurate within 0.2, 0.7, 0.8, and 1.2 percentage points, respectively.

**Fig 6:**
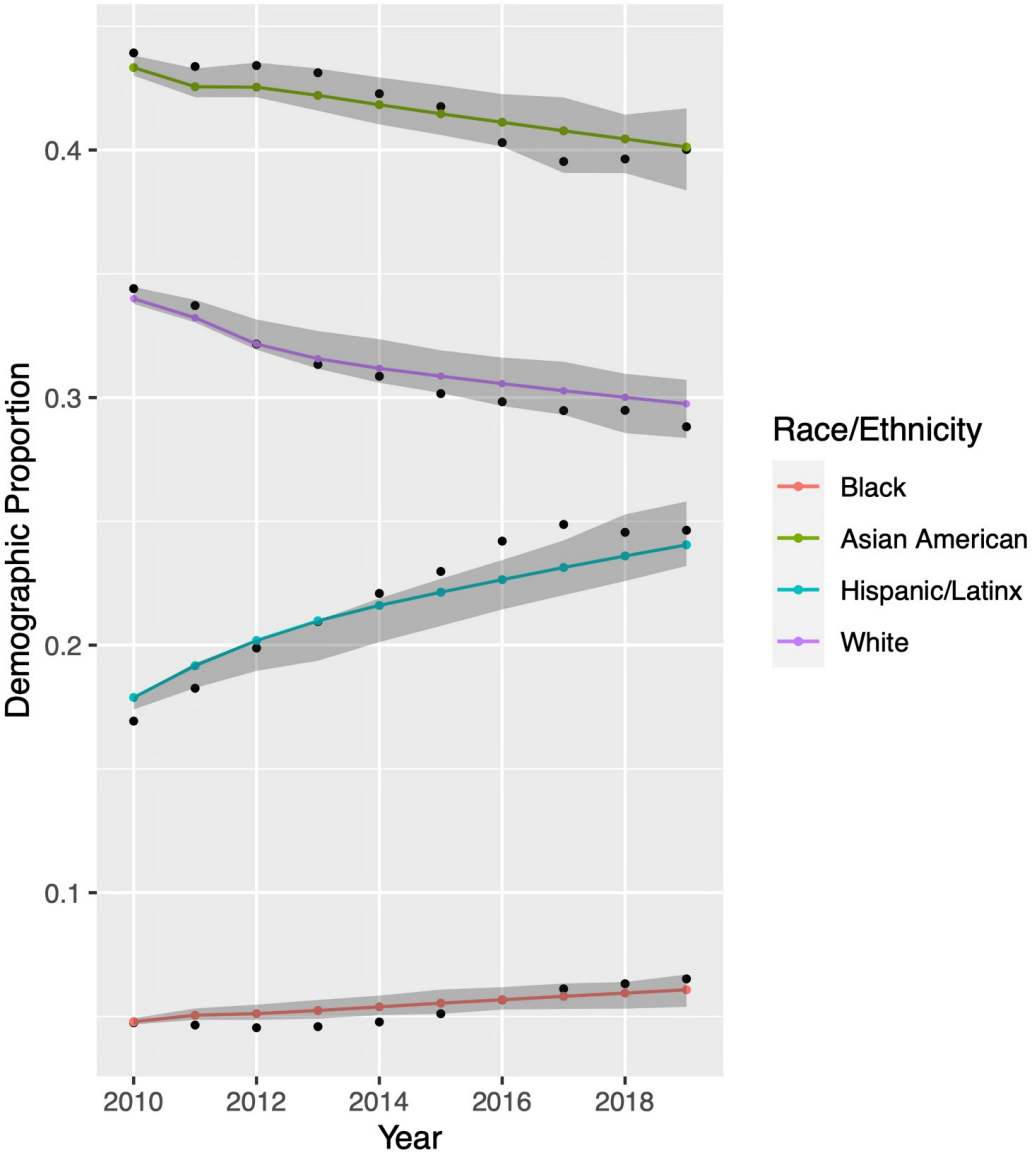
Model validation for University of California-Los Angeles. Historical enrollment records appear as black dots. Colored dots are model output obtained when using the parameters forecast in Inferring Model Parameters. We perform an additional 2800 model simulations using parameter sets generated by Latin hypercube sampling. Grey bands indicate the central 95% of these results. Demographic proportions are relative to each other; see Mathematical Model.

We conduct a similar validation study for UCLA. Using training data that precedes 2010 and historical records from 2010-2019, we find that our model is nearly as accurate with UCLA data as with UCB data. The UCLA results appear in Fig 6.

## Model predictions

**Fig 7:**
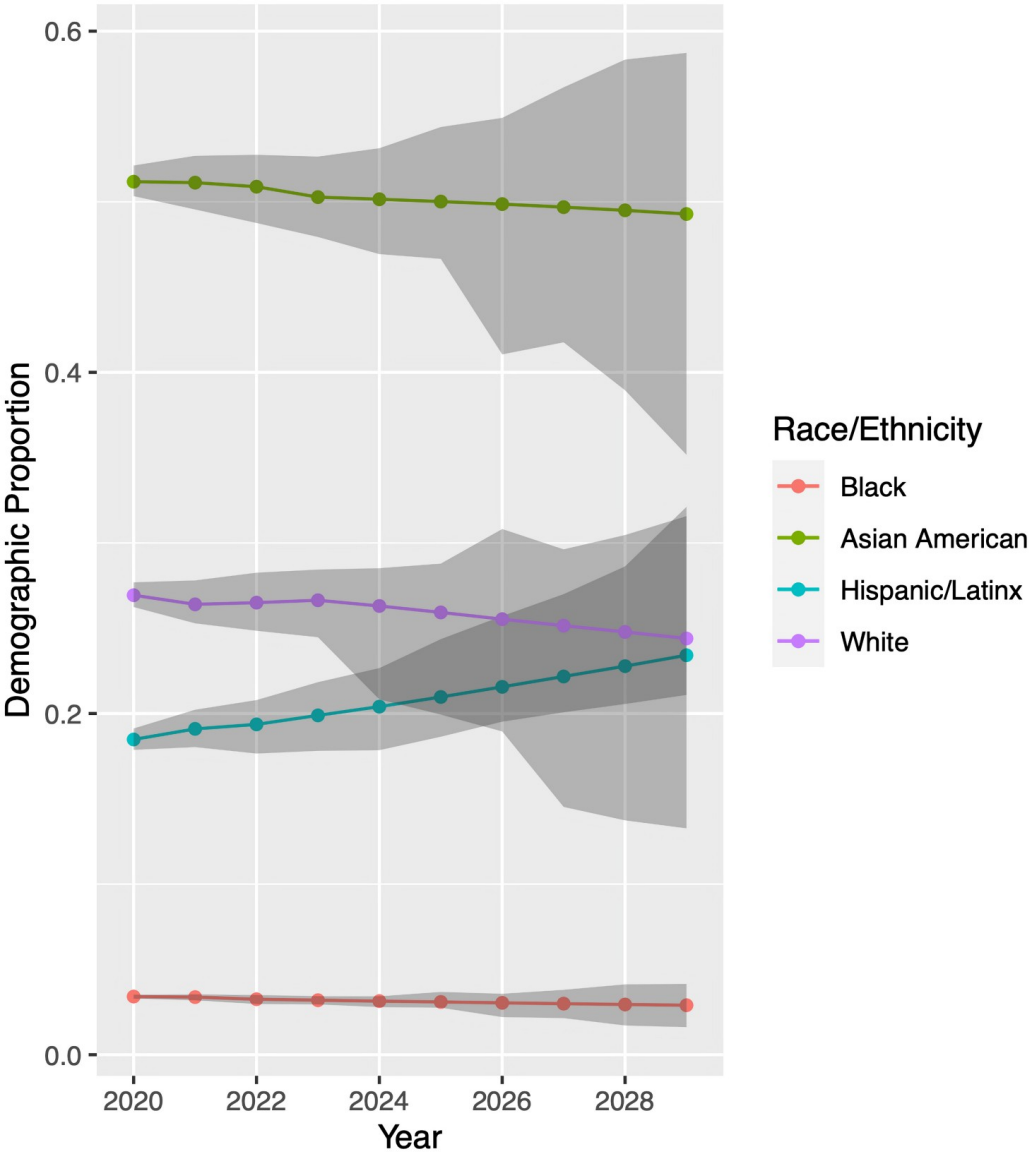
Model predictions for University of California-Berkeley. While the uncertainty in prediction is large after four to five years due to the difficulty in extrapolating model input parameters, two trends are clear. The proportion of Hispanic/Latinx students will rise, and the proportion of Black students will remain quite low. Colored dots are model output obtained when using the parameters forecast in Inferring Model Parameters. We perform an additional 2800 model simulations using parameter sets generated by Latin hypercube sampling. Grey bands indicate the central 95% of these results. Demographic proportions are relative to each other; see Mathematical Model.

**Fig 8:**
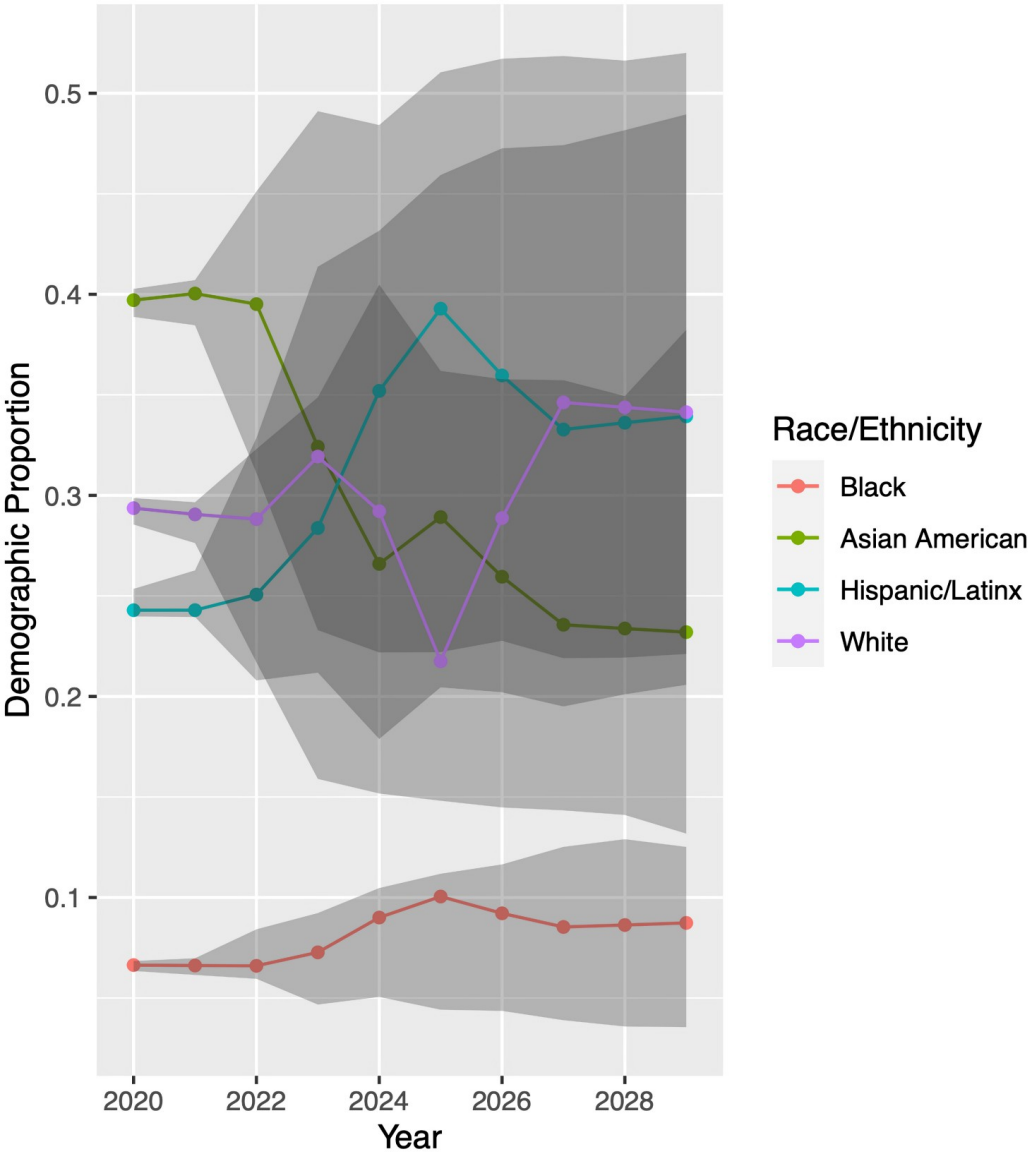
Model predictions for University of California-Los Angeles. While the uncertainty in prediction is large after two years due to the difficulty in extrapolating model input parameters, it is clear that the proportion of Black students will remain quite low. Colored dots are model output obtained when using the parameters forecast in Inferring Model Parameters. We perform an additional 2800 model simulations using parameter sets generated by Latin hypercube sampling. Grey bands indicate the central 95% of these results. Demographic proportions are relative to each other; see Mathematical Model.

We now run our model for each racial/ethnic group to generate predictions for the UCB and UCLA undergraduate student bodies from 2020 through 2029. Results appear in Figs 7 and 8. Again using LHS to randomly sample from within the 95% confidence intervals of our model parameter values, the model projects demographic proportions with a 95% confidence interval, shown in grey.

Fig 7 displays predictions for UCB. These predictions are quite uncertain more than four or five years into the future. The wide confidence bands stem from uncertainty in the extrapolation of model input parameters; see Inferring Model Parameters. Despite the uncertainty in prediction, two trends are clear. The proportion of Hispanic/Latinx students will rise, and the proportion of Black students will remain quite low.

Fig 8 displays predictions for UCLA, which are even more uncertain. Nonetheless, the proportion of Black students will see little growth, if any. The increased uncertainty reflects the poorer performance of models during our input parameter fitting procedure. Although we compared three models for each input parameter, the data sets from UCLA were less well-fit by these models than were those from UCB. A future study could explore other fits for these parameters, which might in turn reduce the uncertainty in our Markov chain model predictions.

## Critical mass

### Defining critical mass

As mentioned in Legal History, the idea of critical mass played a pivotal role in the *Fisher II* Supreme Court case from 2016. According to the court announcement, the University of Texas described critical mass as “an adequate representation of minority students so that the educational benefit that can be derived from diversity can actually happen.” However, the court does not specify what “adequate” and “actually happen” mean.

Recent scholarship has sought to define critical mass. For example, [13] proposes a refinement of critical mass, namely, the concept of *dynamic diversity*. Dynamic diversity comprises four elements, namely, (1) a positive racial climate for each minoritized group; (2) a reckoning with historical exclusion of such groups; (3) equity and inclusion in learning environments; and (4) attention to fostering positive cross-racial interactions. This is a powerful and nuanced model that directly addresses the core goals of affirmative action.

We propose a coarse definition of critical mass. Our definition is much removed from the important goals of dynamic diversity mentioned above. Instead, it is a preliminary step towards operationalizability, and it is directly related to the court’s language of “adequate representation.” We propose that *critical mass of a student demographic group at a college or university is the enrollment level necessary to reflect representation given the institution’s location and available applicant pool*. This definition provides more specificity than the one from *Fisher II* but still allows flexibility. The phrase “given its location and available applicant pool” is necessary in order to allow for the application of critical mass to situations where there are known, purposeful biases in admissions, for example, in-state versus out-of-state admissions for public universities, minority admissions for Historically Black Colleges and Universities (HBCUs), or gendered admissions for single gender institutions. Also, the reference to location recognizes that demographics within the United States are not spatially homogeneous. To forecast critical mass at any institution, we must project national and local demographic data to reflect the school’s applicant pool, and we must account for student residency status to reflect the admissions policy.

### Predicting critical mass

The State of California Department of Finance provides state demographic projections from 2016 until 2060 [14]. Additionally, the U.S. Census Bureau provides population projections from 2016 through 2060 [15]. Both sets of data categorize individuals as ethnically Hispanic (H) or not. Additionally, these data categorize individuals’ race as, among others, Black (B), Asian American (A), or White (W). We combine the racial and ethnic information for these sets of individuals to obtain counts as follows:

$$\begin{array}{c} \text{Black}:B-H\\ \text{Asian}\;\text{American}:A-H\\ \text{Hispanic/Latinx}:(H\cap B)+(H\cap A)+(H\cap W)\\ \text{White}:W\cap H^{c} \end{array}$$

Next, we use the residency demographics of UCB students to determine how many students from each racial/ethnic group come from the in-state applicant pool and the out-of-state applicant pool. UCB only provides this demographic data from 1994 through 2019, so we create our own projections for the residency breakdown for the incoming classes of 2020 through 2029. To do this, based on apparent randomness of the historical data, we use a stochastic process. More specifically, for each respective race/ethnicity-residency status combination, we construct a kernel density estimate from the observed data and draw from it once for each year of projection. We repeat this process 10,000 times and use the mean as our final prediction. We normalize between the in-state and out-of-state residency counts to find the percentage of students for each status.

With these demographic predictions established, we now calculate critical mass. Let us say that *CA*_*ij*_ and *US*_*ij*_ are the proportions that racial/ethnic group *j* makes up in year *i* from the California and U.S. demographic projections, respectively. We will also say that *UCB*_*ij*,in-state_ and *UCB*_*ij*,out-of-state_ are the percents of in-state and out-of-state residents, respectively, in the incoming class at UCB for racial/ethnic group *j* and year *i*. Then we calculate critical mass, *CM*, as

$$\begin{array}{c} CM_{ij}=CA_{ij}\times UCB_{ij,\text{in-state}}+US_{ij}\times UCB_{ij,\text{out-of-state}}. \end{array}$$ (18)

Our critical mass projections appear in Table 3.

**Table 3:** Critical mass projections for University of California-Berkeley. These proportions specify the enrollment level necessary to reflect a racial/ethnic group’s representation given the institution’s location and available applicant pool; see formula in (18). These proportions are relative to each other as our model accounts for only four racial/ethnic groups. This restriction stems from limitations in the availability of data.

	Campus Proportions									
Race/Ethnicity	2020	2021	2022	2023	2024	2025	2026	2027	2028	2029
Black	0.07	0.07	0.07	0.07	0.07	0.07	0.07	0.07	0.07	0.07
Asian American	0.13	0.13	0.13	0.13	0.14	0.14	0.14	0.14	0.14	0.14
Hispanic/Latinx	0.36	0.37	0.37	0.37	0.37	0.38	0.38	0.38	0.38	0.38
White	0.43	0.43	0.43	0.42	0.42	0.42	0.42	0.41	0.41	0.41

### Assessing critical mass

We now compare our UCB student body demographic forecast from Fig 7 to our critical mass predictions from Table 3. This comparison will allow us to assess the degree of inclusion/exclusion of the different racial/ethnic groups. For convenience, we define our critical mass index (CMI) for a given group in a given year as

$$\begin{array}{c} CMI=\frac{\text{Markov}\;\text{chain}\;\text{prediction}}{\text{critical}\;\text{mass}\;\text{prediction}}\times 100. \end{array}$$ (19)

CMI measures how many students we predict will actually enroll at UCB per 100 students that would enroll in an ideally representative class. Furthermore, to provide a 95% confidence interval on our critical mass projections, we compare the middle 95% of critical mass projections to the 95% confidence interval of Markov chain predictions, producing a band of uncertainty on our *CMI* prediction.

**Fig 9:**
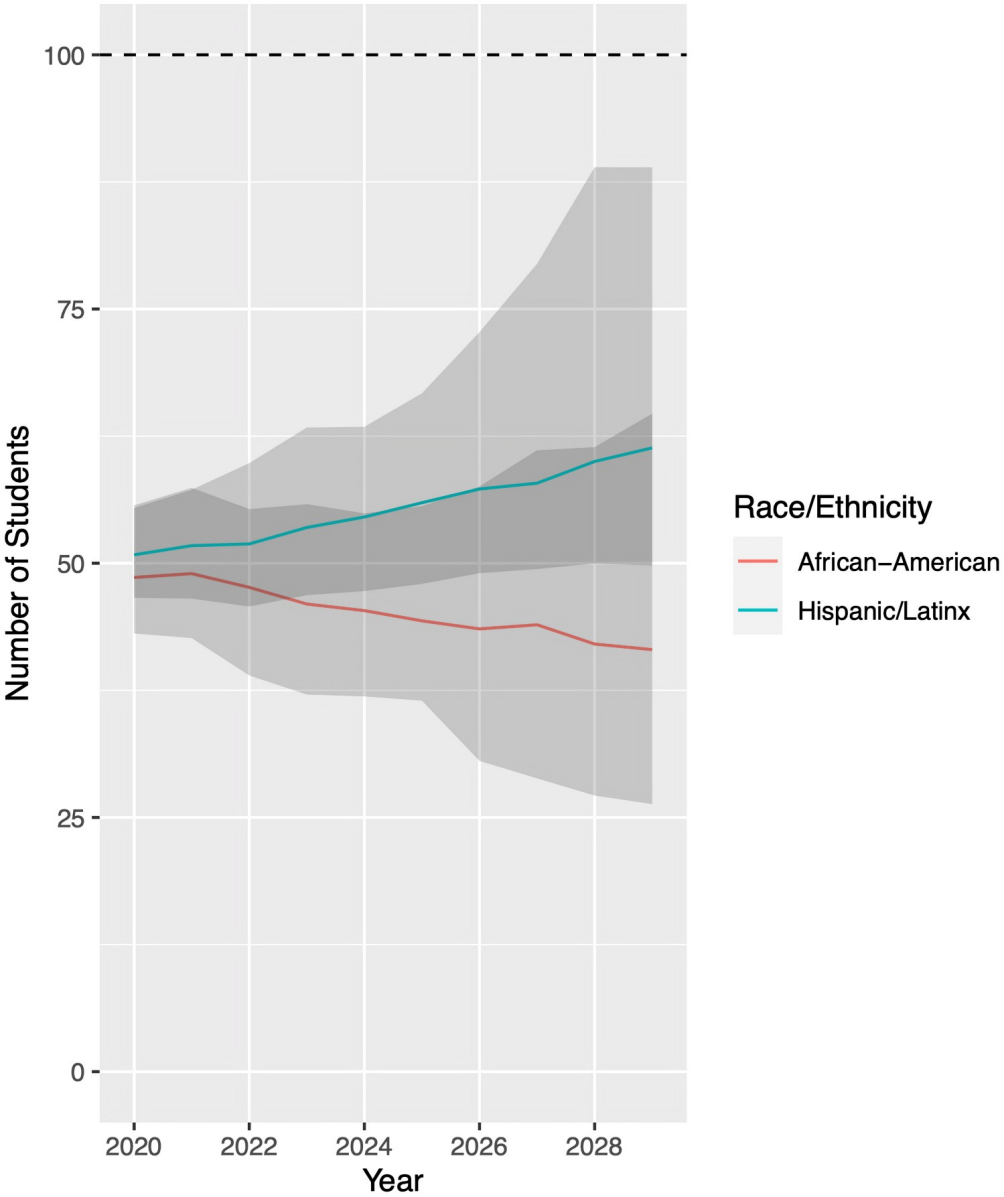
Critical mass index (*CMI*) projections for University of California-Berkeley. For each racial/ethnic group, *CMI* measures the enrollment of students (as predicted by our model) relative to the enrollment of students that would constitute a class representative of the applicant pool, which is defined in Eq (18). For every 100 Black students that UCB should enroll for critical mass, true enrollment will decrease over time (red curve). For every 100 Hispanic/Latinx students that UCB should enroll for critical mass, true enrollment will increase (blue curve). Still, we project that neither group will reach critical mass over the next decade. Grey bands indicate 95% confidence intervals.

Fig 9 plots CMI for the two groups of notable concern. The horizontal line at 100 indicates (theoretically) our Markov chain model predicting the same demographic percentage as our critical mass criterion. Our results demonstrate that Black and Hispanic/Latinx are both underrepresented at UCB. Currently, Black and Hispanic/Latinx groups (coincidentally) make up only half of the proportion that would be representative. Our results predict that under current conditions, Hispanic/Latinx student representation will move towards crtitical mass but will not reach it over the next decade. Black student representation will actually decrease, moving further from critical mass.

## Conclusion

We have constructed a model to forecast student racial/ethnic demographics in bachelor’s degree programs and applied it to two schools in the University of California system, namely, UCB and UCLA. Then, focusing on Black and Latinx students, we proposed a definition for critical mass and used our model to assess whether these student groups would achieve it at UCB campus. We found that Black and Hispanic/Latinx students will continue to be underrepresented, which is consistent with historical trends in higher education.

Implementing an affirmative action policy to rectify these differences is not currently feasible due to Proposition 209, which prohibits public education institutions in California from selecting students on the basis of race, sex, or ethnicity. In November 2020, California voters had the opportunity to vote on Proposition 16, which would have repealed Proposition 209. However, voters rejected the ballot initiative, and so affirmative action remains illegal in California.

While affirmative action in admissions is not a viable strategy for diversifying public campuses in California, there are other well-recognized options. For instance, an admissions office could attempt to recruit more applications from particular racial/ethnic groups. A campus administration could provide more financial, educational, social, and other resources to admitted students in target groups to increase their matriculation rate, and to enrolled students in order to increase their retention rate. What our work adds is the possible quantification of the impact of these strategies. For instance, administrators could use our model to answer questions such as “if we increase the matriculation of Black students by X percentage points per year, they will reach critical mass on our campus in year Y.” While explanations for demographic disparities on campus and the selection of methods for decreasing them are outside the scope of this paper, our model can help identify where, when, and to whom the disparities occur, and with what level of severity.

Our work has important limitations, stemming largely from data availability issues and from simplifications we adopted for the purposes of modeling. For instance, data from the NCES only lists demographic percentages for public school graduates. Data for private schools is not readily available, and so we adopted the same demographic percentages for them, cognizant that fewer than 10% of high school graduates come from these schools. Furthermore, as mentioned earlier, we have not accounted for international students, transfer students, nor students from racial/ethnic groups other than Asian American, Black, Hispanic/Latinx, and White. Even within those four groups, our model ignores complexity by lumping together many disparate racial/ethnic subgroups. Finally, all of the data used in our model was recorded before the COVID-19 pandemic, whose effects we could not account for in our model. We believe the pandemic will significantly impact graduation and admission statistics in the coming years.

Since our model is, to our knowledge, the first one that predicts racial demographics in undergraduate admissions, we sought to make it realistic yet minimal. One possibility for future work is to relax the assumptions mentioned above. Second, we could consider models in which applicants of a particular racial/ethnic group apply, matriculate, and are retained at rates that depend on that group’s representation on campus; essentially, this would be a homophily effect. Third, as we noted with our UCLA predictions, the model’s certainty is strongly dependent on the forecasts of its input parameters. Because our linear models offered a poorer fit for UCLA than for UCB, it would be worthwhile to consider other fits. A fourth possible direction would be to apply our model to universities outside of the University of California system. It would be interesting to discover how universal the model is, and to compare critical mass projections across schools. Fifth, it should be possible to carry out a thorough sensitivity analysis of the model, perhaps using partial rank correlation coefficients or similar methods. Finally, we could investigate whether our modeling framework could be adapted to other axes of diversity—in particular, gender—and to settings other than education, including housing and employment.

We have produced an interactive version of our model as a web app, posted at [16]. This tool allows the user to specify application, admission, and matriculation rates for each racial/ethnic group, and to see demographic projections and critical mass index projections over the next decade. However, due to the computational cost, the public web app does not include uncertainty and confidence interval considerations. This omission increases ease-of-use but we caution that it severely limits predictive capability. Nonetheless, we hope that the interactive tool provides the public with hands-on understanding of our model.
